# Effects of kinesio taping therapy on gait and surface electromyography in stroke patients with hemiplegia

**DOI:** 10.3389/fphys.2022.1040278

**Published:** 2022-11-30

**Authors:** Ze Chen, Min Li, Hongxing Cui, Xipeng Wu, Fangmin Chen, Wei Li

**Affiliations:** ^1^ School of Rehabilitation Medicine, Binzhou Medical University, Yantai, China; ^2^ Department of Rehabilitation, Binzhou Medical University Hospital, Binzhou, China; ^3^ Binzhou Medical University, Yantai, China

**Keywords:** stroke, kinesio taping, sEMG, gait analysis, rehabilitation

## Abstract

**Background:** The application of Kinesio Taping (KT) on the lower extremity of stroke patients can improve the quality of somatosensory information by activating lower extremity muscles involved in postural control. Gait analysis and surface electromyography (SEMG) are valuable in assessing the motor ability of the lower extremities.

**Objective:** This study aimed to investigate the effects of KT therapy on gait and SEMG in stroke patients with hemiplegia.

**Methods:** Twenty-one stroke patients were included in the study. KT was applied to the lower extremities of the hemiplegic side. Quantitative gait parameters were measured by a gait analysis system (IDEEA, by MiniSun, United States) and activation of the lower extremity muscles were evaluated by the SEMG (Trigno™ Wireless Systems, Delsys Inc., United States) before and after taping. Step length, stride length, pulling acceleration, swing power, ground impact, and energy expenditure were used to evaluate when patients walk as usual. SEMG signals were collected from the anterior bilateral tibialis (TA) and the lateral gastrocnemius (LG). The root mean square (RMS) value was used to assess muscle activity. SEMG signals were examined before and after KT treatment in three different locomotor conditions of the patients: walking at a natural speed, walking with a weight of 5 kg, dual-tasking walking (walking + calculation task) while carrying a weight of 5 kg. The calculation task was to ask the patients to calculate the result of subtracting 7 from 100 and continuing to subtract 7 from the resulting numbers. Comparisons between two normally distributed samples (before and after KT treatment) were evaluated using the two-tailed, paired Student’s *t*-test.

**Results:** Stride length (0.89 ± 0.19 vs. 0.96 ± 0.23; *p* = 0.029), pulling acceleration (0.40 ± 0.21 vs. 1.11 ± 0.74; *p* = 0.005), and swing power (0.42 ± 0.24 vs. 1.14 ± 0.72; *p* = 0.004) improved in the hemiplegia side after KT treatment. The RMS value of TA SEMG signals in the limbs on the hemiplegia side decreased after KT treatment during dual-tasking walking carrying a weight of 5 kg (3.65 ± 1.31 vs. 2.93 ± 0.95; *p* = 0.030).

**Conclusion:** KT treatment is effective in altering gait and SEMG characteristics in stroke patients with hemiplegia.

## Introduction

Stroke is a common cerebrovascular event that can result in disabilities including motor, sensory, visual, and cognitive impairments (Ekker et al., 2018). About 70% of stroke survivors exhibit a degree of motor dysfunction that affects daily activities of the patient, social participation, and quality of life (Schröder et al., 2019). Furthermore, in recent decades, an increase of up to 40% has been reported in the incidence of stroke in young adults (Ekker et al., 2018). These young patients have a long-life expectancy after stroke, and the cost of long-term care is a significant burden on healthcare systems. Recovery of motor function on stroke patients is a long and complicated process that requires patients to undergo extensive rehabilitation therapy that involves frequent and regular exercises that match their impairments (Yang et al., 2015). Walking dysfunction is the most common problem in post-stroke patients, and involves an inability to use the ankle dorsiflexor, abnormal gait, and an increased risk of falls due to foot drop (Yang et al., 2015). Falls in stroke patients are closely related to abnormal activation of lower limb muscles (Beyaert et al., 2015; Peishun et al., 2021).

Current common treatment methods for walking rehabilitation include acupuncture, exercise therapy, physiotherapies such as functional electrical stimulation (FES), ankle-foot orthotics (AFO), and kinesio taping (KT) methods (Sheng et al., 2019a). KT is an elastic adhesive tape attached to the surface of the body and is currently used to promote lymphatic circulation, ease pain, provide mechanical support, and improve proprioception (Ekiz et al., 2015). FES can activate the muscles that dorsiflex the ankle and offer an alternative to this treatment for facilitating motor restoration (Sabut et al., 2010). However, electrode placement and instrument manipulation of many FES devices are complex. AFO exert limited effects on walking ability due to increased fatigue and restricted ankle movement (Prenton et al., 2018). In contrast, KT has long been used to strengthen weakened muscles, control muscle tone, improve the active range of motion, balance, functional use, and gait ability as a cost-effective treatment (Park and Bae, 2021). Despite the different treatments available, the mechanisms underlying lower limb muscle activation in stroke patients associated with falls warrants further study.

KT is widely used in the treatment of stroke patients, but the regulation of muscle activation after KT treatment is still unclear. At present, there have been few studies that have objectively evaluated the therapeutic effects of KT on gait stability in stroke patients (Choi et al., 2013; Babyar et al., 2014; Yang et al., 2015). Recently, gait analysis was introduced the ability to measure gait parameters accurately and precisely, allowing clinicians to obtain patient gait information quickly and easily (Gardner et al., 2007). Gait analysis is a well-established tool for the quantitative assessment of gait disturbances, which is helpful to assess the effect of rehabilitation and monitor the disease progress (Baker et al., 2016).

Surface electromyography (SEMG) has the advantages of non-invasion, real-time, and multi-target measurement, and is a method that has received growing attention due to its ability to quantitatively analyze neuromuscular activity in static and dynamic motion states (Rasool et al., 2017). SEMG is a biological electrical signal of the neuromuscular system that is guided and recorded by electrodes placed on the muscle surface (Frigo and Crenna, 2009). SEMG can be used for static muscles, and can observe changes in muscle activity during numerous sports activities (Qie et al., 2020). For example, SEMG has been used to assess normal and abnormal muscle activation in stroke patients and in some patients with sports-injuries, such as those with anterior cruciate ligament injury, to guide rehabilitation strategies (Papagiannis et al., 2019).

KT is an effective treatment for pain caused by sports injury or other diseases, such as osteoarthritis, chronic low back pain, chronic skeletal muscle pain, and delayed onset muscle soreness (Kalinowski and Krawulska, 2017; Hazar Kanik et al., 2019; Rahlf et al., 2019). KT can also improve active range of motion (ROM) and limb function in patients with sports injury (Merino-Marban et al., 2013). Activity during extension movement on taping is conducive to promoting local circulation and improving ROM. In the treatment of stroke patients, the application of KT has been evaluated on shoulder, trunk, upper limb, lower limb and ankle movements, but there have been no studies on the application of KT therapy on the calf and ankle movements simultaneously. SEMG is commonly used to assess muscle function in stroke patients, but is rarely used to assess muscle activation in multiple motor states. In this study, SEMG was used to assess electrophysiological changes in calf muscle activation in stroke patients under three different motor conditions.

Sheng et al. (2019b) found signifcant improvement in stride length, stance phase and swing phase of stroke patients after KT therapy. They suggested that KT may help to improve posture control and instantly produce immediate efects on walking. Jin et al. showed that the study group had significant increases in RMS of vastus medialis SEMG signals after 16 weeks of Baduanjin training (Jin et al., 2017). They also pointed out that RMS of SEMG signals can be used to analyze the recruitment of muscle fibers during contraction. We hypothesized that KT treatment would improve the stride length in stroke patients. The RMS of hemiplegic side TA SEMG signals would increase and RMS of affected side LG SEMG signals wouldn’t change significantly while walking in different locomotor conditions.

## Materials and methods

### Subjects

Twenty-one post-stroke patients with hemiplegia recruited from April 2021 to December 2021 were enrolled in this study. Patients who met the following criteria were included: 1) aged > 30 years; 2) diagnosed with stroke; 3) had a 3–6 months course of stroke; 4) the Brunnstrom stages are at least four; 5) able to walk without assistance; and 6) had foot drop after stroke on the hemiplegic side. Exclusion criteria were as follows: 1) patients with cognitive impairment unable to complete experimental procedures; 2) patients with a history of other neurological diseases or disorders, lower extremity surgery or fracture; or 3) patients exhibiting allergy to KT. At baseline, descriptive variables for each patient, including height, weight and Body Mass Index (BMI), were recorded. All patients were in the middle and late stages of stroke recovery, and their activities of daily living were basically self-care (Barthel index > 60 points). The general characteristics of the subjects are shown in Table 1. The nature and purposes of this study were explained to all participants and all signed an informed consent form prior to participation in the study. The Number of the ethical approval letter is 2021-G 001-01.

**TABLE 1:** Characteristics of the subjects.

Characteristics	Median
Gender (Male/female)	15/6
Age (yrs)	51 (42–57)
Height (cm)	170 (164–172)
Weight (kg)	71.5 (60–78.5)
BMI (kg/m^2^)	24.91 (23.42–27.73)
Type of stroke (Hemorrhage/infarction)	5/16
Affected side (Left/right)	10/11

### Taping intervention

**FIGURE 1:**
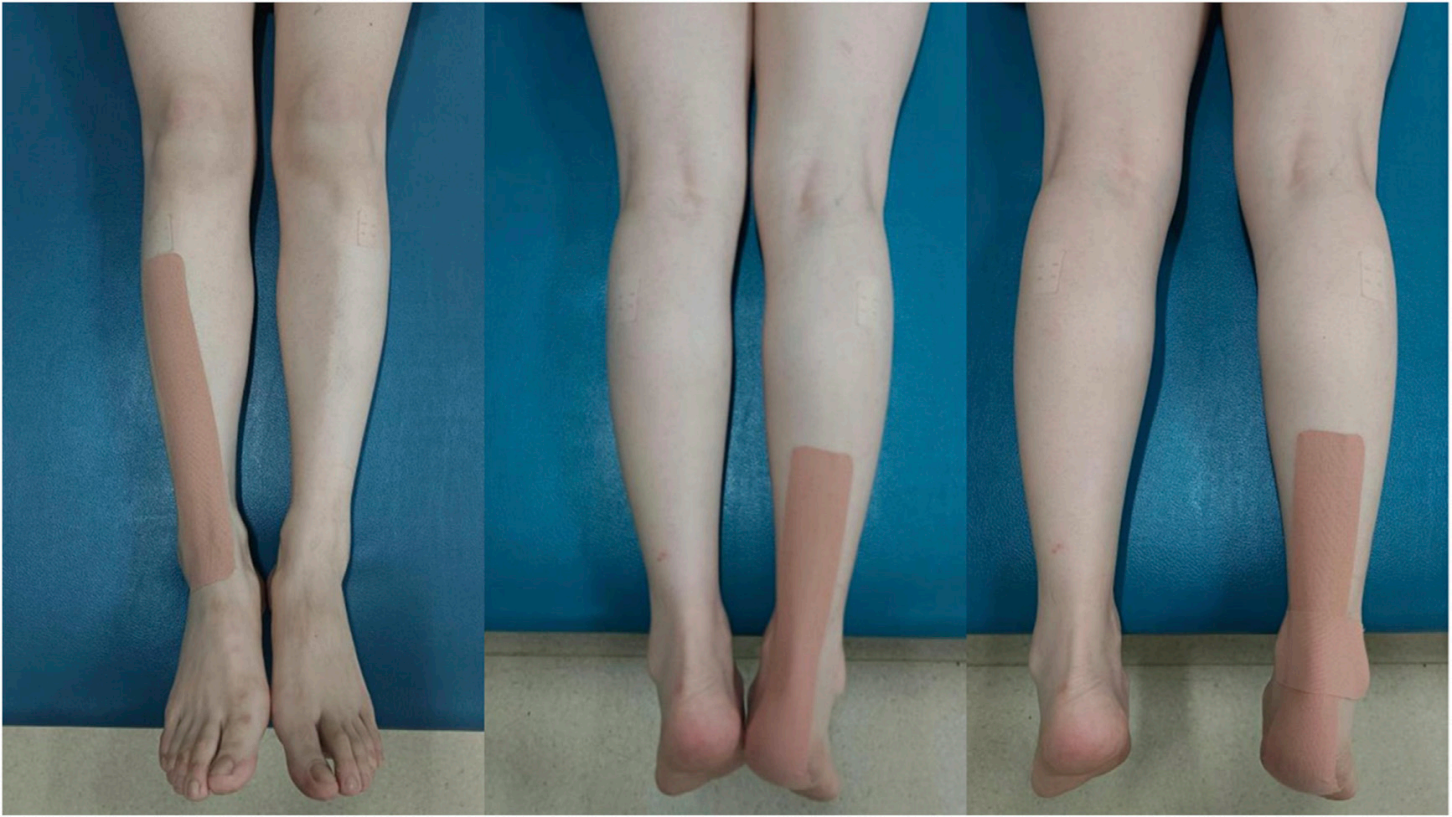
KT taping methods.

Patients underwent routine rehabilitation, including complete training in the hemiplegic limb, exercise training in walking function. All KT treatments were performed by the same qualified physical therapist on the hemiplegic limb of the patient. The area to be taped was cleaned with an alcohol swab prior to KT application. The patients were placed in a supine position during the taping, with their hip, knee, and ankle joints in a neutral position (Rojhani-Shirazi et al., 2015). A kinesio I-shaped strip was placed from the upper end of the external side of the tibia downward along the anterior tibialis muscle and ending at the ankle joint. Two other kinesio I-shaped strips were fixed at the ankle joint in the shape of “x”, where the first was stretched 20-cm from the heel along the Achilles tendon while the other extended from the Achilles tendon to the medial and external malleolus (Figure 1). The pulling force of each kinesio strip was 10% of the maximum tensile length of the tape (Morris et al., 2012; Choi et al., 2013).

### Testing procedures

**FIGURE 2:**
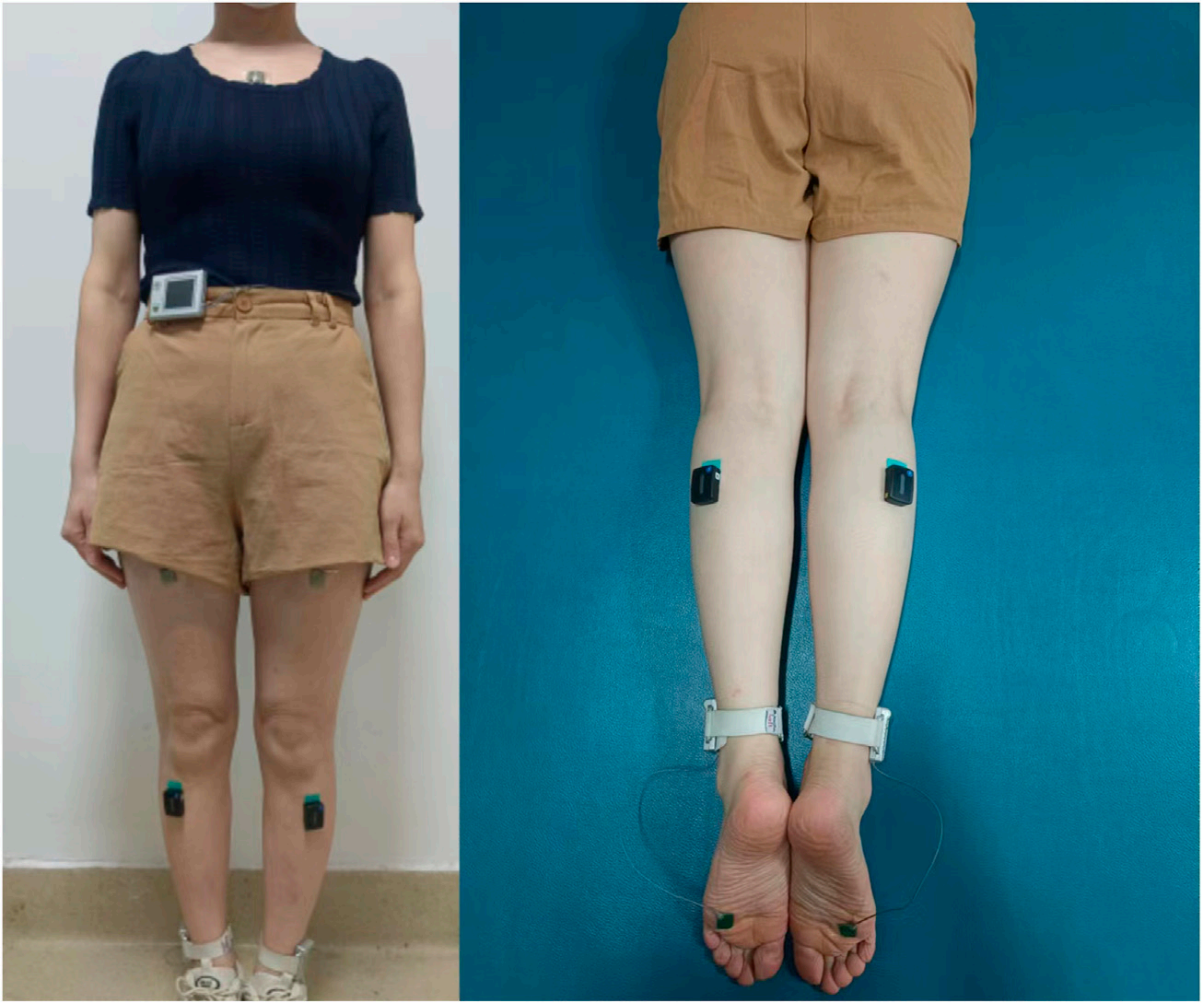
The IDEEA system worn by a patient and the locations of the SEMG electrode sites on the TA and LG.

All evaluations were performed by a qualified physical therapist. Before and after taping, the gait function of the patients was measured using an Intelligent Device for Energy Expenditure and Activity (IDEEA, by MiniSun, United States) device. It is capable of identifying 35 activities and postures and can provide an estimate of energy expenditure when incorporated with basic subject anthropometry (Whybrow et al., 2013) and is capable of both detecting and describing motion as specific activities, including sitting, walking, or stair use, and defining temporal-spatial gait parameters (Mackey et al., 2008). While patients performed the walking exercise, the IDEEA device was used to measure gait parameters including single-limb support time, double-limb support time, swing phase duration, cycle duration, cadence (number of steps per minute), stride length (the distance between the heel points of two consecutive footprints of the same foot), pulling acceleration, swing power, ground impact, foot fall (neuromuscular and skeletal control of the limbs during the end of swing phase), and energy expenditure (EE) (Figure 2). SEMG was measured using a DELSYS wireless dynamic EMG tester (Trigno™ Wireless Systems, Delsys Inc., United States). The bilateral TA and LG SEMG signals were collected and analyzed as these are widely used to evaluate the function of the lower extremity. Subjects walked back and forth under three different locomotor conditions on a 10-m flat floor and the SEMG signals were collected and transmitted *via* a wireless Bluetooth connection. Each parameter was averaged over 20 gait cycles to obtain a representative EMG profile for each muscle. SEMG signals were collected from the TA and LG (Figure 2) under three different locomotor conditions: walking at natural speed, walking holding a weight of 5 kg, and dual-tasking walking (walking + calculation task) holding a weight of 5 kg. For the calculation task, patients were asked to calculate the result of subtracting 7 from 100 and to subtract 7 from the resulting number, and so on. Evaluations were conducted before and after KT taping immediately. All measurements were collected twice for each patient (Sheng et al., 2019a).

### Signal processing

The SEMG signals were processed and analyzed using EMGworks Analysis and MATLAB R2020a (MathWorks, 3 Apple Hill Dr, Natick, MA 01760-2098). The sampling frequency was 1,200 Hz. The root mean square (RMS) value was analyzed and used to evaluate muscle activity. TA and LG muscles were intermittently activated during the test. SEMG data was collected continuously throughout the test. The signals for each dorsal extension and plantar flexion of the ankle were isolated for further analysis. We combined SEMG data from 20 gait cycles using SEMG analysis software. SEMG synchronization points were subjected to analysis using MATLAB software after noise elimination. First, the raw SEMG signals were digitally zero-phase filtered by a second-order Butterworth bandpass filter (20–450 Hz) and then they were full-wave rectified. Second, the RMS values of the full-wave rectified signals were computed within a 50-ms time window. The baseline SEMG activity was determined to be six times the standard deviation of the first 50 ms of the RMS values, where there was no contraction in the dorsal extension and plantar flexion of the ankle muscles. RMS values greater than the baseline were detected and used to determine the beginning and ending of the dorsal extension and plantar flexion of ankle epochs. The difference between the indices of these values was calculated. The point where the difference between the two indices is greater than 250 ms was identified as the end of an epoch. In accordance with de Jesus et al. (2010), the rectified SEMG signals in the identified epochs greater than the baseline values were defined as effective myoelectric activation (EMA) of the muscles.

### Statistical analysis

All the data was analyzed in SPSS software (Version 26.0, IL, United States). Data are reported as mean ± standard deviation (M ± SD). The Shapiro–Wilk normality test was performed to examine the normality distribution of the data. Comparisons between two normally distributed samples (before and after KT treatment) were evaluated using the two-tailed, paired Student’s *t*-test. A *p*-value < 0.05 was considered statistically significant.

## Results

### Gait measurement results

Stride length [before: 0.89 ± 0.19 (m); after: 0.96 ± 0.23 (m); *p* = 0.029], pulling acceleration [before 0.40 ± 0.21 (G); after: 1.11 ± 0.74 (G); *p* = 0.005], and swing power [before: 0.42 ± 0.24 (G); after: 1.14 ± 0.72 (G); *p* = 0.004] all increased after taping when patients were performing walking activities (Table 2).

**TABLE 2:** Comparison of the gait parameters before taping and after taping for patients (*N* = 21) walking at a normal pace.

	Before taping	After taping	
Variables	(M ± SD)	(M ± SD)	*p*-value
Step length (m)	0.44 ± 0.10	0.48 ± 0.12	0.190
Stride length (m)	0.89 ± 0.19	0.96 ± 0.23	0.029*
Cadence (steps/min)	87.38 ± 23.04	92.06 ± 20.31	0.138
Speed (steps/sec)	0.69 ± 0.30	0.75 ± 0.30	0.122
Single support phase (%)	38.62 ± 4.04	36.31 ± 4.63	0.255
Double support phase (%)	15.68 ± 6.66	16.55 ± 7.33	0.715
Pulling acceleration (G)	0.40 ± 0.21	1.11 ± 0.74	0.005*
Swing power (G)	0.42 ± 0.24	1.14 ± 0.72	0.004*
Ground impact (G)	0.80 ± 0.45	0.85 ± 0.42	0.242
EE (kcal/m)	3.24 ± 1.10	3.19 ± 0.95	0.843

*Indicates significant differences (*p* < 0.05).

### Surface electromyography measurements

SEMG was measured during three walking modes (walking, walking carrying a weight of 5 kg, and dual-tasking walking carrying a weight of 5 kg and calculation tasks simultaneously) for all patients. The RMS of the hemiplegic side TA SEMG signals significantly decreased (before: 3.65 ± 1.31; after: 2.93 ± 0.95; *p* = 0.030) after KT treatment during dual-tasking walking carrying a weight of 5 kg but not for other modes of walking (Table 3).

**TABLE 3:** Comparison of affected-side muscles SEMG signals before taping and after taping when patients performed three distinct walking modes.

Variables	TA (M ± SD)			LG (M ± SD)		
RMS (V)	Before taping	After taping	*p*-value	Before taping	After taping	*p*-value
Walking at a natural speed	3.71 ± 1.37	3.16 ± 1.13	0.086	2.59 ± 0.86	2.85 ± 1.25	0.333
Walking with a weight of 5 kg	3.64 ± 0.93	3.70 ± 1.50	0.782	2.62 ± 1.02	2.91 ± 1.29	0.285
Dual-tasking walking with a weight of 5 kg	3.65 ± 1.31	2.93 ± 0.95	0.030*	2.47 ± 0.95	2.87 ± 1.28	0.233

*Indicates statistical significance (p < 0.05).

## Discussion

In this study, we found that KT could increase stride length, pulling acceleration, and swing power (*p* < 0.05) in affected limbs, improve calf muscle strength, and improve gait stability when patients walked at natural speed. This result is consistent with previous hypothesis that KT treatment would improve the stride length in stroke patients. Furthermore, we also found the RMS value of the affected side TA SEMG signals were different from LG muscles. The RMS value of affected side LG SEMG signals increased after KT taping, but there was no statistical significance (*p* > 0.05). The RMS value of the affected side TA SEMG signals decreased (*p* < 0.05) after KT treatment when the patients performed dual-tasking walking carrying a weight of 5 kg. This result is inconsistent with previous hypothesis that the RMS of hemiplegic side TA SEMG signals would increase and RMS of affected side LG SEMG signals would decrease while walking in different locomotor conditions.

Gait parameters such as stride length, pulling acceleration and swing power were improved after KT therapy. We attributed these findings to the fact that strengthening the TA muscle and mechanical support of the KT contributes to better ankle control. Kase et al. have reported that KT increases muscle activation through the following two mechanisms (Ekiz et al., 2015). Firstly, KT stimulates cutaneous receptors by tactile stimulation and increases sensory input to recruit more motor units during the most vigorous contraction of the muscle (Chang et al., 2010). Secondly, KT increases the subcutaneous volume and blood flow (Akbaş et al., 2011). In addition, studies have shown that walking ability and efficiency of the patient improved immediately after the KT treatment (Ekiz et al., 2015; Choi et al., 2016; Sheng et al., 2019b). Lee and Bae (2021) demonstrated the short-term effect of application of lower-leg KT according to the proprioceptive neuromuscular facilitation (PNF) pattern increased the gait ability of chronic stroke patients with foot drop. KT application has also been found to have a more positive effect on stance phase duration than McConnell taping in patients with stroke (Sung et al., 2017). Park et al. compared the immediate effects of talus stabilization taping (TST) with those of KT on the ankle dorsiflexion passive range of motion (DF-PROM), static balance ability, the Timed Up and Go (TUG) test, and fall risk in patients with chronic stroke (Park and Lee, 2019). Further, the TUG results decreased significantly in the KT group. These results are consistent with a previous study (Sheng et al., 2019a). Sheng et al. (2019a) demonstrated that the ankle KT intervention significantly improved the 10-m walking test, the TUG results, the stride length, the stance phase, and the swing phase in patients with a foot drop after stroke. The application of viscoelasticity and continuous mechanics of KT can also support and stabilize muscles and joints, deepen sensory input and promote circulation that reduces pain and swelling (Koseoglu et al., 2017).

In the study, the RMS value of the affected side TA SEMG signals decreased (*p* < 0.05) after KT treatment in stroke patients while performing multiple tasks synchronously. We could speculate that when patients performed multitask walking synchronously, the activation of the affected side TA muscle decreased. We considered that the patient should pay closer attention on multitasking, which affected the precision of the walking movement (de Barros et al., 2021). Walking ability is related to cognitive functions such as executive function and attention (O'Brien and Holtzer, 2021; Ueda et al., 2018). Executive function is a series of high-level functions involved in the processing of information from the posterior cortical sensory system to the anterior cortex to produce the corresponding movements, including the intention or start of action (walking), decision-making and control behavior. Attention is a dynamic function driven by sensory perception that requires selecting a primary stimulus for a particular behavior (walking) while ignoring unnecessary and irrelevant stimuli. Studies have also shown that due to neural damage affecting postural control, greater cognitive resources must be mobilized during walking, leading to a high cognitive load in stroke patients when dealing with dual tasks. Postural control motor dysfunction also increases the demand for limited attention resources (Yang et al., 2018; Koren et al., 2022).

The cognitive function of stroke patients tends to decline. Cognitive-motor and motor dual tasks play important roles in daily life, such as walking while talking, using a mobile phone, carrying a bag, or watching traffic (Yang et al., 2007). Previous studies have indicated that performing two tasks simultaneously can negatively impact on gait performance. Dual task interference that affects gait performance has been observed not only in healthy subjects, but also in subjects with neurological disorders (Yang et al., 2007). In stroke-injured individuals, reductions in speed, cadence, and stride length, as well as increases in stride time during cognitive-motor dual tasking have been reported. In addition, stroke subjects have more difficulty performing dual motor tasks compared to healthy adults (O Brien and Holtzer, 2021). Diminished capacity for dual task performance and reduced ability to adapt to changing environments may limit the ability of individuals with stroke to return to the community. Consequently, improving walking ability in dual task situations is an important goal, especially for subjects experiencing chronic stroke with limited ambulation in the community setting (Lu et al., 2015).

Furthermore, gait analysis and SEMG technology were used to provide an objective and quantitative method for evaluating the effect of KT therapy. Approximately 70% of patients with hemiplegia recover their ability to walk after these therapies but retain abnormal gait and problems such as foot drop and pronation (Gandolla et al., 2018). These abnormal movement patterns affect stability, walking safety, and consume more energy than a regular gait (Cho et al., 2020). It remains a difficult problem to improve gait ability in the legs of patients with hemiplegia in rehabilitation therapy (Park and Lee, 2016). Furthermore, an objective and quantitative evaluation of the rehabilitation effect is also the trend and requirement of precision rehabilitation therapy (Babyar et al., 2014; Wonsetler and Bowden, 2017; Lerma Castaño et al., 2020). However, most studies use scales and experimental tests, such as the modified Ashworth Scale (MAS), the Stroke-Specific Quality of Life Scale (SS-QLS), and the 10-m walk test (10 MWT); these assessments can be affected by subjective factors (Mohan et al., 2021). The combination of gait analysis and SEMG can quantitatively evaluate the amount of rehabilitation experienced by patients and thus provide an objective reference for rehabilitation programs (Zhu et al., 2019; Peishun et al., 2021).

Moreover, we observed improvement in the stability of the ankle joint and improvement of the high tension of the extensor muscle when walking using a taping formula based on the plasticity theory of the central nervous system (Park and Bae, 2021). As such, continuous sensory input was similar to the continuous correction of deviation by a therapist (Hu et al., 2019). Compared to traditional one-to-one rehabilitation treatment, patients detect correct motion guidance more directly when using KT (Santos et al., 2019). Clinicians can also observe a patient’s gait and muscle activation with a gait analyzer and SEMG, which allows for adjustments to the rehabilitation treatment plan in real time (Choi et al., 2016).

This study demonstrates that KT treatment can improve gait in stroke patients with hemiplegia when patients do not perform multiple tasks simultaneously. However, some limitations to the present study should also be considered. First, this study did not conduct a hierarchical comparison study that included multiple types of stroke or tested different KT taping methods as the etiopathological mechanism(s) and the clinical presentation of stroke is complex (Lee et al., 2017). We also need to further explore changes in brain function during multitasking. Furthermore, the lasting effects of the application of KT therapy are unknown due to the small number of samples and the lack of long-term follow-up observation; thus, additional research is necessary to establish the duration and efficacy that KT provides.

## Conclusion

KT therapy is effective in improving gait ability and muscle function among stroke patients with hemiplegia, KT therapy helps prevent falls and promotes recovery among stroke patients with hemiplegia.

## Data Availability

The original contributions presented in the study are included in the article/supplementary material, further inquiries can be directed to the corresponding authors.
